# Line-of-Duty Deaths Among Firefighters in Poland: A Retrospective Observational Study of Mortality Differences Between Career and Volunteer Firefighters

**DOI:** 10.3390/jcm15124616

**Published:** 2026-06-14

**Authors:** Kamil Pająk, Marcin Gruchała, Jakub Sobolewski, Andrzej R. Reindl

**Affiliations:** 1 Department of Environmental Toxicology, Faculty of Health Sciences with the Institute of Maritime and Tropical Medicine, Medical University of Gdańsk, 80-204 Gdańsk, Poland; kamil.pajak@gumed.edu.pl; 2 First Department of Cardiology, University Clinical Center, Medical University of Gdańsk, 80-214 Gdańsk, Poland; marcin.gruchala@gumed.edu.pl (M.G.); jakub.sobolewski@gumed.edu.pl (J.S.)

**Keywords:** firefighters, fatalities, line-of-duty death, LODD, occupational health, occupational exposure, Poland

## Abstract

**Background:** Firefighting is a hazardous occupation, yet data online-of-duty deaths in European firefighter populations remain limited. This study aimed to characterise the mechanisms and circumstances of firefighter fatalities in Poland and to estimate exposure-based fatality rates, with particular attention to differences between career and volunteer personnel. **Methods:** In this retrospective observational study, line-of-duty firefighter fatalities in Poland from 1995 to 2025 were identified from a nationwide repository and cross-validated against official sources. The mechanism of death was classified from case narratives following the Utstein framework. Group comparisons used chi-square, Fisher’s exact, and Welch’s tests; multivariable probit regression assessed predictors of mechanism; and per-capita and per-deployment fatality rates were computed using national denominator data. **Results:** Of 112 fatalities, 73 (65.2%) involved volunteer firefighters. Sudden Cardiac Arrest of Presumed Non-Traumatic origin (SCA-PNT) was the leading mechanism (44.6%), followed by traumatic injury (37.5%). Volunteers were older than career firefighters (46.4 ± 14.0 vs. 34.6 ± 8.7 years; *p* < 0.001) and more likely to die of SCA-PNT (odds ratio 6.35; 95% confidence interval 2.46–16.40) and during the response phase (odds ratio 5.07; 1.89–13.55). Age was the strongest independent predictor of mechanism. The per-capita fatality rate was higher among career firefighters (incidence rate ratio 5.16), whereas the per-deployment rate was higher among volunteers (incidence rate ratio 2.25). **Conclusions:** Firefighter mortality in Poland differs by employment status and is strongly age-dependent. Age-stratified cardiovascular surveillance and prevention may be more effective than formation-based approaches.

## 1. Introduction

In Poland, the operational firefighting workforce eligible to participate in rescue and firefighting operations comprises approximately 29,000 career (full-time) firefighters and 280,000 volunteer (on-call) firefighters. Firefighting is an inherently hazardous occupation. Operational duties and training activities involve exposure to a unique constellation of acute and chronic hazards—including strenuous physical exertion, high ambient temperatures, exposure to combustion-derived pollutants, risk of structural collapse, and acute psychological stress—which together carry a substantial risk of injury and mortality [1,2,3,4,5,6]. The intensity and nature of these exposures vary according to incident characteristics, operational environment, and assigned role within the organizational structure [4,7].

In the United States and other Western countries, the leading cause of line-of-duty deaths among firefighters is sudden cardiac death (SCD)—typically defined and adjudicated according to National Institute for Occupational Safety and Health (NIOSH) or United States Fire Administration (USFA) criteria—which accounts for approximately 45–50% of all duty-related fatalities [2,8,9,10,11,12,13]. Recent analyses indicate that traumatic injuries are the second leading cause, accounting for approximately 27% of fatalities, while combined asphyxiation and burn injuries account for roughly 20% [14,15,16].

In the European context, and particularly in Central and Eastern Europe, occupational exposures and line-of-duty fatalities among firefighters remain insufficiently investigated. The present study focuses on Polish firefighters—both career personnel and volunteers. Given the limited availability of prior research addressing the causes and circumstances of firefighter fatalities in Poland, the present study aimed to (i) characterize the profile and circumstances of line-of-duty firefighter fatalities in Poland over a 31-year period, with comparison between career and volunteer personnel, and (ii) estimate fatality rates per capita and per operational deployment using national State Fire Service (PSP) statistical bulletins as denominator data. Beyond the Polish context, the analytical framework—particularly the use of operational deployment data as a denominator—may be applicable to other fire services facing similar epidemiological challenges.

## 2. Materials and Methods

### 2.1. Study Setting and Data Sources

A retrospective observational study of line-of-duty fatalities among firefighters in Poland was conducted for the period from 1 January 1995, to 31 December 2025. The observation window was set to begin in 1995, coinciding with the establishment of the National Firefighting and Rescue System (Krajowy System Ratowniczo-Gaśniczy, KSRG), which fundamentally reorganized firefighting operations in Poland and marked the beginning of the contemporary structure of the Polish fire service.

In accordance with the Polish Act of 4 April 2014 on Compensation Benefits in the Event of Accidents or Diseases Related to Service [17], a line-of-duty fatality was defined as a sudden event resulting in death occurring during the performance of official duties or in direct connection with such duties. Eligible events included emergency response operations (fire and non-fire incidents), responding to or returning from incidents, participation in mandatory training or physical fitness activities, and other officially assigned tasks such as equipment maintenance or inspections.

Cases were ascertained from the FireTrap repository (https://firetrap.pl/polegli-na-sluzbie: accessed on 12 January 2026), an independent, publicly accessible nationwide platform documenting Polish firefighter line-of-duty deaths. It is acknowledged that FireTrap is not an integrated government administrative registry; nevertheless, at the time of analysis it represented the most comprehensive longitudinal source of firefighter mortality data publicly available in Poland, as no equivalent national registry is maintained by the State Fire Service or the Ministry of the Interior. To mitigate the risk of incomplete or inaccurate reporting, each FireTrap entry was cross-checked against three independent source types: (i) official communiqués issued by the National Headquarters of the State Fire Service (Komenda Główna Państwowej Straży Pożarnej, KG PSP) and its regional voivodeship-level commands; (ii) contemporaneous reporting in regional and national media; and (iii) firefighting periodicals and online portals, including Przegląd Pożarniczy (published by the KG PSP), W Akcji (published independently by Elamed Media Group), and the firefighting portals remiza.pl and strazacki.pl.

Aggregate statistics on the operational activity of the Polish Fire Service—including the annual number of incidents (fires, non-fire emergencies, and false alarms) and the annual number of firefighter participations in operations, stratified by formation (career vs. volunteer)—were retrieved from the annual statistical bulletins published by the National Headquarters of the State Fire Service (KG PSP). Bulletins for the years 2005–2024 are publicly available online (https://www.gov.pl/web/kgpsp/biuletyny-informacyjne-psp---roczne: accessed on 12 January 2026), whereas data for 1995–2004 were obtained from the printed bulletins held in the library of the Fire University (Akademia Pożarnicza) in Warsaw. Aggregate data for 2025, not yet published in bulletin form, were obtained from the official communications of the National Headquarters of the State Fire Service.

### 2.2. Data Extraction and Outcome Classification

Each reported line-of-duty fatality was reviewed by two researchers, who jointly examined the source narrative and any available corroborating materials. Classifications were assigned by consensus, with uncertain cases referred to a third researcher for adjudication. Extracted variables included firefighter age, sex, and employment status (career or volunteer), date of death, incident type (fire, non-fire incident, or non-emergency), duty stage at the time of death (on-scene operations, training, response to or return from incidents, or other duties), and mechanism of death. Where available, additional descriptive information was recorded to identify recurrent contextual factors associated with fatalities, including motor vehicle crashes and deaths occurring while donning personal protective equipment (PPE).

#### 2.2.1. Classification of Mechanism of Death

Because medical records and autopsy findings were not available, classification of the mechanism of death relied exclusively on narrative case descriptions, supplemented by official statements where available. Each fatality was assigned to one of seven mutually exclusive categories based on the most proximate identifiable mechanism: (A) traumatic injury, defined as death caused by mechanical force (motor vehicle accident, structural collapse, fall from height, explosion, or being struck by an object); (B) asphyxiation or inhalation injury; (C) drowning; (D) electrocution; (E) thermal injury or burns; (F) sudden cardiac arrest of presumed non-traumatic origin (SCA-PNT); and (G) other or unspecified causes, including anaphylactic shock, choking, and aspiration pneumonia. This taxonomy broadly corresponds to the “medical” (category F) and “traumatic/exposure-related” (categories A–E) groupings used in NIOSH and USFA firefighter fatality reports [18], although the present classification preserves greater granularity within the latter group.

#### 2.2.2. Adjudication of Difficult Cases

In eight cases—all occurring during structural fires—the primary mechanism of death could not be unambiguously determined from the available narratives, as multiple mechanisms (inhalation injury, thermal injury, and mechanical trauma) potentially contributed. Adjudication followed a consistent decision rule: cases were assigned to traumatic injury (category A) when the narrative or an official statement from the KG PSP explicitly described injuries consistent with mechanical trauma (explosion, structural collapse, or fall through a structure) as the proximate cause of death, and to asphyxiation/inhalation injury (category B) when the firefighter remained inside a burning structure without evidence of primary mechanical injury. Applying this rule, five cases were assigned to traumatic injury and four to asphyxiation/inhalation injury. The implications of this adjudication framework for the distribution of mechanisms across formations are addressed in the Limitations section.

#### 2.2.3. Classification of SCA-PNT

Classification of category F followed the Utstein recommendations for the reporting of out-of-hospital cardiac arrest [19]. A case was assigned to this category when the narrative indicated sudden collapse during or shortly after duty, with cardiopulmonary resuscitation attempted, and in the absence of any identifiable traumatic, asphyxial, drowning, thermal, or electrical mechanism. The term “cardiac arrest” is used in its mechanistic sense—cessation of mechanical cardiac activity—rather than as an etiological diagnosis. Because autopsy findings and clinical records were not available, this category is presumed to comprise predominantly cardiovascular events but may also include non-cardiac causes of sudden collapse, such as pulmonary embolism, intracranial hemorrhage, aortic dissection, or hypoglycemia. The term “sudden cardiac death” was deliberately avoided, as it would imply a confirmed cardiac etiology unavailable in this dataset; similarly, the term “non-cardiac death” was avoided because it incorrectly suggests that cardiac arrest is exclusively cardiac in origin.

For binary comparative analyses, the seven categories were collapsed into two principal groups: deaths with an identifiable external mechanism (categories A–E, n = 59) and SCA-PNT (category F, n = 50). Category G (n = 3) is reported descriptively but excluded from binary comparisons due to small numbers and etiological heterogeneity.

### 2.3. Statistical Analysis

#### 2.3.1. Descriptive Statistics and Group Comparisons

Descriptive statistics were used to characterise the study population. Categorical variables were summarised as counts and percentages, and continuous variables were reported as means with standard deviations or medians with interquartile ranges, as appropriate.

Comparisons of categorical variables between career and volunteer firefighters were performed using the chi-square test of independence when expected cell counts allowed. When expected cell counts were small, Fisher’s exact test was used for 2 × 2 tables, and the Fisher–Freeman–Halton exact test was applied to larger contingency tables; for tables in which exact computation was not feasible, *p*-values were obtained by Monte Carlo approximation with 50,000 replicates. Mean differences in age between independent groups were assessed using Welch’s t-test, which does not assume equal variances. To quantify the strength of associations between binary exposures and outcomes, odds ratios (ORs) with 95% confidence intervals (CIs) were calculated using the normal approximation of the log-odds; reference categories are specified in the text and table footnotes. Comparisons of mechanism distribution by age were conducted using a 50-year cut-point selected a priori. This threshold is defined in Polish regulations governing periodic medical examinations of operationally active volunteer firefighters: the Regulation of the Minister of Health of 11 April 2022 (Journal of Laws, item 828) stipulates examinations every 2–3 years up to age 50, and every 1–2 years thereafter [20]. To examine whether the association between employment status and mechanism of death persisted after accounting for age and other covariates, a multivariable probit regression was fitted, with mechanism of death as the binary outcome (SCA-PNT versus identifiable external mechanism; category G excluded). Covariates comprised age, year of incident, employment status, duty stage, and incident type. Overall model fit was summarised using McFadden’s pseudo-R^2^, and the joint contribution of the non-age covariates was assessed by a likelihood-ratio test comparing the full model with an age-only model. A logistic-regression specification examined as a sensitivity analysis yielded consistent conclusions.

#### 2.3.2. Calculation of Fatality Rates

To estimate the occurrence of line-of-duty fatalities in relation to the underlying exposure of the Polish fire service, two complementary fatality rates were computed: a per-capita rate, expressed per 100,000 firefighter-years, and a per-deployment rate, expressed per 1,000,000 firefighter-deployments. The per-capita rate uses the size of the operational workforce as the denominator and quantifies the risk borne by an individual firefighter over a year of service. The per-deployment rate uses the number of individual firefighter participations in operational incidents as the denominator and quantifies the risk associated with a single operational deployment. The two rates are complementary: career firefighters participate in many more operational incidents per year than volunteers, so the two metrics can yield divergent comparisons between formations, each addressing a different occupational health question.

Aggregate numbers of incidents and of firefighter participations in operations (stratified by formation, career vs. volunteer) for the period 1995–2025 were obtained from the annual statistical bulletins of KG PSP, as described in Section 2.1. Internal consistency of the denominator data was verified by confirming that the annual sum of fires, non-fire emergencies, and false alarms equalled the reported total number of incidents for every year of the observation window. For the per-capita denominator, the operational workforce was treated as a constant point estimate of approximately 29,000 career firefighters (PSP) and 280,000 operationally eligible volunteer firefighters (OSP) for the full observation period. These figures were obtained directly from KG PSP in official written communication and corroborated by publicly available datasets published under a Creative Commons license on the National Open Data Portal (https://dane.gov.pl/pl/dataset/4695,statystyki-zdarzen-swdpsp-2024: accessed on 12 January 2026) for 2024. The assumption of relative temporal stability of the workforce is justified by the statutory regulation of PSP staffing levels, and is addressed further in the Limitations section. Fatality rates and their 95% confidence intervals were computed under a Poisson model; incidence rate ratios (IRRs) between formations were computed as the ratio of formation-specific rates, with 95% confidence intervals derived from the standard error of the log-IRR.

#### 2.3.3. Software

All statistical analyses were performed using IBM SPSS Statistics (version 29.0; IBM Corp., Armonk, NY, USA) and Gretl (version 2024a). Two-sided *p*-values < 0.05 were considered statistically significant.

## 3. Results

### 3.1. Overall Characteristics

A total of 112 line-of-duty firefighter fatalities were identified in Poland between 1995 and 2025, of which 73 (65.2%) occurred among volunteer firefighters (OSP) and 39 (34.8%) among career firefighters (PSP). The cohort was overwhelmingly male (109/112, 97.3%); the three female fatalities (4.1% of OSP, 0.0% of PSP) all occurred in the volunteer subgroup. Fatalities were distributed across the 31-year observation window with a median of four cases per year (range 1–9), and three calendar years (2000, 2008, and 2014) recorded no fatalities.

The age at death ranged from 18 to 69 years (overall mean ± SD: 42.3 ± 13.6 years; median 42, IQR 32–54). Volunteer firefighters who died in the line of duty were on average more than a decade older than career firefighters: 46.4 ± 14.0 years (median 50, IQR 37–57) among volunteers versus 34.6 ± 8.7 years (median 34, IQR 29–39) among career firefighters (Welch’s t-test, t = 5.45, *p* < 0.001; see Section 2.3.1).

### 3.2. Mechanism of Death

The distribution of mechanisms of death across the seven Utstein-derived categories is summarised in Table 1. Sudden cardiac arrest of presumed non-traumatic origin (SCA-PNT) was the single most frequent mechanism overall, accounting for 50 of 112 fatalities (44.6%), followed by traumatic injury (42/112, 37.5%), drowning (9/112, 8.0%), asphyxiation or inhalation injury (4/112, 3.6%), electrocution (3/112, 2.7%), thermal injury (1/112, 0.9%), and other or unspecified causes (3/112, 2.7%).

The distribution of mechanisms differed markedly between formations (global comparison of the 7 × 2 contingency table, Fisher–Freeman–Halton exact test with Monte Carlo approximation; see Section 2.3.1: *p* < 0.001). Among volunteer firefighters, SCA-PNT was the leading mechanism (43/73, 58.9%), followed by traumatic injury (23/73, 31.5%). Among career firefighters, this pattern was reversed: traumatic injury was the leading mechanism (19/39, 48.7%), followed by SCA-PNT (7/39, 17.9%). Career firefighters also bore the entire burden of asphyxiation or inhalation injuries (4/4 cases), and two-thirds of all drowning deaths (6/9), reflecting the typical operational profile of internal structural firefighting and water rescue.

In a binary comparison restricted to SCA-PNT (category F) versus identifiable external mechanisms (categories A–E), and excluding the heterogeneous category G (n = 3), the relative odds of SCA-PNT versus an identifiable external mechanism were 6.35 times higher in volunteer firefighters than in career firefighters (odds ratio = 6.35, 95% CI 2.46–16.40; Fisher’s exact test, *p* < 0.001; reference category: career firefighters; see Section 2.3.1 for methodology). An OR greater than 1 thus indicates higher relative odds of SCA-PNT, rather than an external mechanism, among volunteer firefighters compared with career firefighters.

The age profile of fatalities differed substantially by mechanism. The mean age at death was 51.2 ± 10.6 years for SCA-PNT (median 52) and 34.7 ± 11.4 years for the combined external-mechanism categories A–E (median 33); the difference was assessed using Welch’s t-test (see Section 2.3.1) and was highly statistically significant: t = 7.78, *p* < 0.001. The age distribution of mechanism subgroups (Table 1, footnote) suggested clinically coherent patterns: SCA-PNT cases were concentrated in the oldest age range, traumatic-injury cases at intermediate age, and electrocution cases at the youngest ages (mean 20.0 ± 1.7 years, n = 3). Within the SCA-PNT subgroup, volunteer firefighters were on average more than a decade older than career firefighters with SCA-PNT (53.0 ± 9.5 vs. 39.7 ± 11.0 years; Welch’s t-test, *p* = 0.018), although the small number of career SCA-PNT cases (n = 7) warrants cautious interpretation.

### 3.3. Duty Stage and Incident Type

Operational characteristics of fatal incidents are summarised in Table 2. The distribution of duty stages at the time of death differed significantly between formations (global comparison of the 4 × 2 contingency table, Fisher–Freeman–Halton exact test with Monte Carlo approximation, 50,000 replicates; see Section 2.3.1: *p* < 0.001).

Among volunteer firefighters, fatalities occurred predominantly during the response to or return from an incident (35/73, 47.9%), followed by on-scene operations (33/73, 45.2%); training accounted for a small minority of cases (5/73, 6.8%), and no volunteer fatalities were recorded during other officially assigned duties. Among career firefighters, this distribution was reversed: on-scene operations were the leading duty stage at the time of death (21/39, 53.8%), followed by training, responding to or returning from incidents, and other duties (each 6/39, 15.4%). The relative odds of death during the response or return phase, compared with all other duty stages combined, were more than five times higher in volunteer firefighters than in career firefighters (OR = 5.07, 95% CI 1.89–13.55; Fisher’s exact test, *p* < 0.001; reference category: career firefighters; see Section 2.3.1). The disaggregated counts indicated that this excess was driven almost entirely by the responding phase (34 volunteer vs. 3 career fatalities) rather than the returning phase (1 vs. 3, respectively), suggesting that the elevated risk concentrates before—not after—the operational task. Of the 35 volunteer fatalities recorded during responding or returning, six occurred specifically during donning of personal protective equipment, all classified as SCA-PNT.

Conversely, the relative odds of death during officially assigned duties unrelated to emergency response (the “other” category, including equipment maintenance, inspections, and similar tasks) were substantially lower in volunteer firefighters than in career firefighters (OR = 0.04, 95% CI 0.00–0.64; Fisher’s exact test, *p* = 0.001; reference category: career firefighters; Haldane–Anscombe correction applied due to a zero cell). All six fatalities during “other” duties occurred in career personnel.

The distribution of incident types at which fatalities occurred also differed between formations (Fisher–Freeman–Halton exact test, *p* = 0.005). Volunteer fatalities clustered in operational incidents—fires (38/73, 52.1%) and non-fire emergencies (27/73, 37.0%)—with only a small proportion (8/73, 11.0%) occurring during non-emergency activities. Among career firefighters, the distribution was more even, and non-emergency activities accounted for as many fatalities as fires (14/39, 35.9% each), followed by non-fire emergencies (11/39, 28.2%). The relative odds of death during non-emergency activities, compared with operational incidents combined, were nearly five times lower in volunteer firefighters than in career firefighters (OR = 0.22, 95% CI 0.08–0.59; Fisher’s exact test, *p* = 0.003; reference category: career firefighters). The relative odds did not differ significantly between formations for fire incidents (OR = 1.94, 95% CI 0.87–4.31; *p* = 0.116) or non-fire emergencies (OR = 1.49, 95% CI 0.64–3.47; *p* = 0.406).

### 3.4. Age Distribution

The detailed distribution of fatalities by age group and employment status is presented in Table 3. The overall distribution differed significantly between formations (global comparison of the 6 × 2 contingency table, Fisher–Freeman–Halton exact test with Monte Carlo approximation, 50,000 replicates; see Section 2.3.1: *p* < 0.001). The age structure of volunteer fatalities was skewed towards older ages, with the largest single age group being 50–59 years (26/73, 35.6%) and 52.1% (38/73) of volunteer fatalities occurring at age 50 years or older. In contrast, career firefighter fatalities clustered in the third and fourth decades of life, with 76.9% (30/39) occurring before age 40 and no fatalities recorded at age 60 years or older.

#### 3.4.1. Age Distribution of SCA-PNT Fatalities

**Figure 1:**
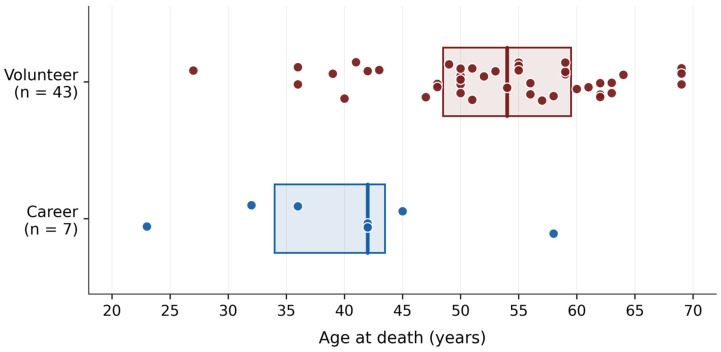
Age at death of SCA-PNT fatalities (n = 50). Each dot represents one case. Solid line: median; box: IQR. Volunteer (n = 43): median 54, IQR 49–60, range 27–69. Career (n = 7): median 42, IQR 34–44, range 23–58. Welch’s t-test, t = 3.03, *p* = 0.018.

A more pronounced age-related pattern emerged when the analysis was restricted to the SCA-PNT subgroup (n = 50; Figure 1). The single largest age group was 50–59 years, accounting for 42.0% (21/50) of all SCA-PNT cases, followed by 40–49 years (22.0%, 11/50) and the ≥60 years group (22.0%, 11/50). Overall, 64.0% (32/50) of SCA-PNT cases occurred in firefighters aged 50 years or older. This pattern was particularly marked among volunteer firefighters, in whom 46.5% (20/43) of SCA-PNT cases occurred at 50–59 years and a further 25.6% (11/43) at 60 years or older—notably, all of the latter falling within the age range at which volunteer firefighters remain legally eligible to participate in rescue operations under Polish regulations (up to 65 years for general operational duty, with selected exemptions for drivers above that age) [REF: Polish Act on Volunteer Fire Brigades, 17 December 2021, Art. 9]. Among the seven career firefighters who died of SCA-PNT, the age distribution was shifted to younger ages, with 42.9% (3/7) occurring at 40–49 years and only one case at 50 years or older. Volunteer SCA-PNT decedents were on average more than a decade older than their career counterparts (medians 54 vs. 42 years; Welch’s t-test, t = 3.03, *p* = 0.018; see Section 2.3.1).

#### 3.4.2. Age-Stratified Comparison of Mechanisms

Within the cohort of fatalities attributable to an identifiable mechanism (categories A–F, n = 109; category G excluded due to small numbers), SCA-PNT was markedly concentrated in firefighters aged 50 years or older, whereas fatalities from external mechanisms occurred predominantly in younger firefighters (Table 4). Only 18 of 68 fatalities in the under-50 group (26.5%) were attributable to SCA-PNT, compared with 32 of 41 fatalities (78.0%) in the 50-and-over group. The relative odds of SCA-PNT versus an external mechanism were approximately ten times higher among firefighters aged 50 years or older than among those younger than 50 (OR = 9.88, 95% CI 3.96–24.66; Fisher’s exact test, *p* < 0.001; reference category: firefighters younger than 50 years; see Section 2.3.1).

### 3.5. Fatality Rates

Aggregate denominator data for the period 1995–2025 are summarised in Table 5. Over the 31-year observation window, the Polish State Fire Service recorded a total of 12,937,637 incidents (4,362,617 fires, 7,813,790 non-fire emergencies, and 761,230 false alarms), to which career firefighters contributed a total of 46,628,000 individual participations and volunteer firefighters 38,732,000. With operational workforce point estimates of 29,000 career and 280,000 operationally eligible volunteer firefighters (Section 2.3.2), this corresponds to an average of approximately 51.9 operational participations per year for a career firefighter and 4.5 for a volunteer firefighter—an 11.6-fold difference in mean per-capita operational exposure.

The two complementary fatality rates yielded contrasting comparisons between formations (Table 5). Expressed per 100,000 firefighter-years, the per-capita rate was substantially higher among career firefighters (4.34, 95% CI 3.09–5.93) than among volunteers (0.84, 95% CI 0.66–1.06), corresponding to an incidence rate ratio of 5.16 (95% CI 3.50–7.61, *p* < 0.001; reference category: volunteer firefighters; see Section 2.3.2). Expressed per 1,000,000 firefighter-deployments, the per-deployment rate was conversely lower among career firefighters (0.84, 95% CI 0.60–1.14) than among volunteers (1.89, 95% CI 1.48–2.37), with an incidence rate ratio of 2.25 (95% CI 1.53–3.32, *p* < 0.001; reference category: career firefighters). Combined across formations, the overall per-capita fatality rate was 1.17 per 100,000 firefighter-years (95% CI 0.96–1.41), and the overall per-deployment fatality rate was 1.31 per 1,000,000 firefighter-deployments (95% CI 1.08–1.58).

The opposite directions of these two incidence rate ratios reflect the markedly different operational exposure patterns of the two formations and underscore the importance of denominator choice in the interpretation of occupational mortality. The substantially higher per-capita rate among career firefighters indicates that, over a year of service, an individual career firefighter faces a substantially greater absolute probability of a fatal occupational event than an individual volunteer; the higher per-deployment rate among volunteers indicates that a given operational participation carries a higher probability of a fatal outcome for a volunteer than for a career firefighter. Neither metric alone fully characterises occupational risk, and both warrant consideration in the design of preventive strategies.

When the analysis was restricted to the SCA-PNT subgroup (n = 50), the per-capita rates of SCA-PNT were similar between formations (0.78 per 100,000 firefighter-years for career firefighters, 95% CI 0.31–1.60; 0.50 for volunteers, 95% CI 0.36–0.67; incidence rate ratio 1.57, 95% CI 0.71–3.49, *p* = 0.27), whereas the per-deployment rate of SCA-PNT was approximately seven-fold higher among volunteers (1.11 per 1,000,000 deployments) than among career firefighters (0.15 per 1,000,000 deployments). This pattern is consistent with the interpretation that SCA-PNT risk in volunteer firefighters is concentrated in the acute exposure of individual operational events—including the pre-operational alarm response and PPE-donning phases described in Section 3.3—rather than in the cumulative workload of routine service.

## 4. Discussion

### 4.1. Principal Findings

This 31-year nationwide analysis of line-of-duty firefighter fatalities in Poland yielded three principal findings. First, sudden cardiac arrest of presumed non-traumatic origin (SCA-PNT) was the leading mechanism of death overall (44.6%) and the dominant mechanism among volunteer firefighters, while traumatic injury predominated among career firefighters. Second, the two formations differed markedly in the operational circumstances of death: volunteer fatalities occurred predominantly while responding to or returning from incidents, whereas career firefighter fatalities were concentrated in on-scene operations and training. Third, the choice of denominator fundamentally altered the comparison of occupational risk between formations—career firefighters had a substantially higher per-capita fatality rate, whereas volunteers had a higher per-deployment rate—underscoring that occupational mortality in this population cannot be meaningfully summarised by a single metric.

### 4.2. Mechanism Profile, Predictors, and the Importance of Denominator Choice

The predominance of non-traumatic sudden death among on-duty firefighter fatalities in Poland is consistent with the broad consensus in the international literature. Sudden cardiac events account for approximately 45–50% of on-duty firefighter deaths in the United States, with traumatic injuries representing the second leading cause [2,6,9], the overall mechanism distribution observed in the present cohort closely parallels these figures. As in several other national series, non-traumatic sudden death in our cohort was concentrated among volunteer firefighters and during the alarm-response phase [4,8,14,21].

A central observation of this study concerns the contrasting per-capita and per-deployment fatality rates between formations. Expressed per 100,000 firefighter-years, the fatality rate was more than five-fold higher among career firefighters than among volunteers (incidence rate ratio 5.16); expressed per 1,000,000 deployments, the rate was conversely more than two-fold higher among volunteers (incidence rate ratio 2.25). This apparent paradox is explained by the markedly different operational exposure of the two formations: an individual career firefighter participated, on average, in approximately 11.6 times as many operational incidents per year as an individual volunteer. The two rates address different questions—the per-capita rate reflects the cumulative annual risk borne by an individual, while the per-deployment rate reflects the risk attached to a single operational event—and both are necessary for a balanced interpretation of occupational risk. This finding directly addresses a key limitation of fatality counts that are not normalised to underlying exposure, and cautions against interpreting the numerical preponderance of volunteer deaths as evidence of higher individual risk in that group.

To examine whether the bivariate association between employment status and mechanism of death persisted after accounting for age, we conducted supplementary multivariable analyses (probit regression; SCA-PNT versus identifiable external mechanism, n = 109, category G excluded). Age was by far the strongest predictor of mechanism of death (probit coefficient = 0.073 per year, *p* < 0.001), and alone accounted for the majority of the explained variance (McFadden pseudo-R^2^ 0.300 for the age-only model versus 0.390 for the full model). After adjustment for age, employment status retained a smaller but statistically significant residual association (coefficient = −0.875, *p* = 0.031; career firefighters being less likely to die of SCA-PNT than volunteers of the same age), as did the training duty stage (coefficient = 1.785, *p* = 0.044). The addition of all operational and demographic covariates to the age-only model produced only a marginal, non-significant improvement in fit (likelihood-ratio test, *p* = 0.058). Taken together, these results indicate that the older age structure of the volunteer cohort is the principal driver of its higher proportion of SCA-PNT deaths, while a smaller residual effect of formation—potentially reflecting differences in fitness, health surveillance, or operational conditions—persists after adjustment. From a preventive standpoint, this reframes the at-risk group: rather than targeting volunteers as a category, age-stratified cardiovascular risk assessment for firefighters aged 50 years or older of either formation may represent the more rational intervention.

The operational circumstances of fatal events differed substantially between formations and offer a coherent physiological interpretation. Among volunteers, fatalities occurred predominantly during the response to or return from incidents, whereas career firefighter deaths were more frequently recorded during on-scene operations and training. A substantial proportion of SCA-PNT events occurred outside direct fireground involvement: approximately 48% of all SCA-PNT deaths were associated with the alarm response, transit, or donning of personal protective equipment (PPE), with a further 24% occurring during non-emergency activities, including training. This distribution is consistent with the abrupt sympathetic and hypothalamic–pituitary–adrenal activation that accompanies alarm response, producing rapid surges in heart rate and blood pressure that may precipitate an acute coronary event in a susceptible individual [22,23,24].

The concentration of volunteer fatalities in the pre-operational phase, in contrast to the on-scene and training concentration among career firefighters, likely reflects the structural differences between the two systems. Volunteer firefighters typically respond to an alarm from rest—often from home or work—and must rapidly mobilise, travel to the fire station, don PPE, and proceed to the incident, compressing intense adrenergic and physical stress into a short pre-operational window. Career firefighters, by contrast, begin duty shifts already at the station, are physically prepared for deployment, and consequently encounter their highest-risk exposures during the operational and training activities that constitute the core of their shift-based service. This interpretation aligns with the observation that all six donning-related fatalities in the cohort—each classified as SCA-PNT and occurring during the response phase—were among volunteers.

### 4.3. Occupational Stressors and Non-Traumatic Fatalities

Firefighters are exposed to a wide range of harmful occupational factors. These include toxic combustion products, high ambient temperatures, extreme physical exertion, psychological stress, disruption of circadian rhythm, and work in oxygen-depleted environments [1,2]. Strenuous physical activity, emotional stress, and environmental pollutants all strain the cardiovascular system, and each can increase the risk of sudden cardiac events in susceptible individuals [2,25,26,27]. These stressors can promote dehydration, blood clotting, and inflammation, which over time may damage the arteries and, acutely, precipitate a coronary event. Cardiac events are more likely to occur in firefighters who already have underlying disease, such as coronary atherosclerosis or structural heart disease, in whom an acute occupational stressor can act as a trigger [6,28].

Importantly, exposure to demanding fireground conditions represents only a small part of a firefighter’s duties, yet a disproportionate share of non-traumatic deaths occurs outside active fire suppression. In our cohort, many non-traumatic sudden deaths occurred during the alarm, response, and PPE-donning phases—that is, before arrival at the scene [2,28]. This finding supports the view that these early phases carry substantial risk: the alarm itself may trigger an abrupt adrenergic stress response, with a rapid increase in heart rate and blood pressure driven by sympathetic activation and catecholamine release, often before the firefighter has even reached the vehicle [2,6,22].

Two factors are particularly relevant to the Polish context. First, exposure to toxic combustion products is compounded by background air pollution: Poland has one of the highest burdens of air-pollution-related premature mortality in Europe [29], so firefighters here may carry an additional, chronic cardiovascular burden on top of their occupational exposures. Second, retired career firefighters in Poland commonly continue to serve as volunteers. Some individuals therefore accumulate occupational exposure across decades of combined professional and volunteer service which, together with advancing age, may help explain the older age profile and the higher proportion of non-traumatic deaths observed in the volunteer group. We could not assess this directly in our data, but it is a plausible contributing factor.

### 4.4. Implication for Prevention

Because age was the strongest predictor of non-traumatic death, preventive efforts should be guided by age rather than by formation alone: age-stratified cardiovascular risk assessment for firefighters aged 50 years or older, of either formation, may be more rational than treating volunteers as a single high-risk group. This is especially important given the asymmetry in health surveillance in Poland—career firefighters undergo comprehensive annual examinations and periodic fitness testing, whereas volunteers are examined less frequently, across a narrower scope, and undergo no mandatory fitness testing. Older volunteers thus carry the highest burden of non-traumatic death yet are the least closely monitored.

The content of screening also warrants reconsideration. Resting electrocardiography, the mainstay of current protocols, often fails to detect the non-obstructive but unstable plaques that may rupture under acute haemodynamic stress. More sensitive tools—coronary artery calcium scoring, coronary computed tomographic angiography, and high-sensitivity cardiac troponin (hs-cTn)—can identify subclinical disease beyond traditional risk factors [30,31]. Individual risk factors should also be managed, including overweight and obesity, hypertension, dyslipidaemia, atherosclerosis, and diabetes mellitus as part of the obesity-related disease spectrum. Beyond screening, evidence-based pharmacotherapy may help: SGLT2 inhibitors (empagliflozin, dapagliflozin) reduce sudden cardiac death in patients with diabetes, heart failure, or chronic kidney disease [32], and fixed-dose “polypill” strategies improve long-term adherence to cardiovascular therapy [33]. For individuals at high but transient arrhythmic risk, a wearable cardioverter-defibrillator may provide temporary protection [32,34]. Equally important is education: raising awareness among firefighters of the risk factors for non-traumatic sudden death—and of the warning signs and the value of regular health monitoring—is a low-cost measure that may improve early recognition and encourage participation in preventive care.

Finally, standard sudden-cardiac-death risk scores may perform poorly here. Derived in older general-population cohorts, they rely heavily on overt disease—heart failure, prior myocardial infarction, diabetes, atrial fibrillation [35]—precisely what occupational screening tends to exclude from active service. A firefighter may therefore appear “low risk” yet harbour subclinical coronary disease that turns lethal only under acute occupational stress, arguing for risk assessment tailored to occupationally active, apparently healthy populations.

### 4.5. Strengths and Limitations

This study has several strengths. To our knowledge, it is the first comprehensive analysis of line-of-duty firefighter fatalities in Poland, and one of only a few such studies in Europe, spanning more than three decades following the establishment of the KSRG. By examining both career and volunteer firefighters, and by classifying the mechanism of death according to the Utstein recommendations, it provides a structured account of duty-specific risk patterns that allows meaningful comparison with international evidence. A particular strength is the calculation of exposure-based fatality rates: by combining fatality counts with national denominator data on workforce size and operational deployments, we were able to express risk on both a per-capita and a per-deployment basis, rather than relying on absolute counts alone.

Several limitations should be acknowledged. Most importantly, the dataset does not originate from an official, centralised national registry but from a publicly accessible repository of narrative incident descriptions. Although every case was cross-checked against official communiqués and other independent sources, misclassification of duty stage or mechanism of death cannot be fully excluded, and incomplete case ascertainment remains possible. Because medical records and autopsy findings were not available, the mechanism of death—particularly SCA-PNT—was inferred from narrative descriptions rather than confirmed pathologically; this category is therefore presumed, not proven, to be predominantly cardiac in origin. The absence of individual clinical data also meant that cardiovascular risk factors and prior health status could not be assessed. Finally, the fatality rates rely on workforce estimates treated as constant over the study period and on aggregate deployment counts, and should therefore be interpreted as approximations; they do not account for individual variation in exposure, task allocation, or time in active service. Despite these limitations, the consistency of the findings with international evidence, together with the rigorous cross-referencing of sources, supports their validity in the absence of a formal national surveillance system.

## 5. Conclusions

This 31-year nationwide analysis indicates that line-of-duty firefighter mortality in Poland differs by employment status, with non-traumatic sudden deaths concentrated among older volunteer firefighters and traumatic deaths more common among younger career firefighters. Age appeared to be the principal factor associated with the mechanism of death, while the apparent effect of formation was largely, though not entirely, explained by the older age structure of the volunteer group. The contrasting per-capita and per-deployment fatality rates further suggest that occupational risk in this population cannot be captured by a single measure and depends on the denominator considered.

These findings should be interpreted with caution, given the retrospective design and the absence of medical confirmation of the cause of death. Nonetheless, they point to several directions that may merit consideration: more consistent and age-appropriate health surveillance across both formations, the use of more sensitive cardiovascular screening tools in older firefighters, and broader preventive measures, including risk-factor management and education. Prospective studies that incorporate individual health data and precise exposure metrics would help to confirm these observations and to establish which preventive strategies are most effective in this occupational group.

## Figures and Tables

**Table 1:** Mechanism of death by employment status in Polish line-of-duty firefighter fatalities.

Mechanism of Death	Volunteer (n = 73)	Career (n = 39)	Total (n = 112)
A. Traumatic injury	23 (31.5)	19 (48.7)	42 (37.5)
B. Asphyxiation/inhalation injury	0 (0.0)	4 (10.3)	4 (3.6)
C. Drowning	3 (4.1)	6 (15.4)	9 (8.0)
D. Electrocution	2 (2.7)	1 (2.6)	3 (2.7)
E. Thermal injury/burns	1 (1.4)	0 (0.0)	1 (0.9)
F. SCA-PNT	43 (58.9)	7 (17.9)	50 (44.6)
G. Other/unspecified ^a^	1 (1.4)	2 (5.1)	3 (2.7)

Values are presented as n (column %). Mechanism of death was classified based on the most proximate mechanism described in case narratives; category F (SCA-PNT) followed the Utstein recommendations for out-of-hospital cardiac arrest, as detailed in Methods Section 2.2. Overall distribution differed significantly between groups (Fisher–Freeman–Halton exact test, Monte Carlo approximation with 50,000 replicates: *p* < 0.001). Binary comparison of SCA-PNT (F) vs. identifiable external mechanism (A–E): odds ratio = 6.35 (95% CI 2.46–16.40); reference category: career firefighters; Fisher’s exact test, *p* < 0.001; methodology described in Section 2.3.1. Category G excluded from the binary comparison. ^a^ Category G comprises one case of anaphylactic shock, one of choking, and one of aspiration pneumonia.

**Table 2:** Duty stage and incident type at the time of line-of-duty fatality, by employment status.

	Volunteer (n = 73)	Career (n = 39)	OR (95% CI) ^a^	*p* ^b^
Duty stage				
On-scene operations	33 (45.2)	21 (53.8)	0.71 (0.32–1.54)	0.431
Training	5 (6.8)	6 (15.4)	0.40 (0.11–1.42)	0.187
Responding or returning ^c^	35 (47.9)	6 (15.4)	5.07 (1.89–13.55)	<0.001
Other duties	0 (0.0)	6 (15.4)	0.04 (0.00–0.64) ^d^	0.001
Incident type				
Fire	38.(52.1)	14 (35.9)	1.94 (0.87–4.31)	0.116
Non-fire emergency	27 (37.0)	11 (28.2)	1.49 (0.64–3.47)	0.406
Non-emergency activity	8 (11.0)	14 (35.9)	0.22 (0.08–0.59)	0.003

Values are presented as n (column %). Overall distribution differed significantly between formations for both duty stage and incident type (Fisher–Freeman–Halton exact test with Monte Carlo approximation, 50,000 replicates: *p* < 0.001 and *p* = 0.005, respectively; see Section 2.3.1 for methodology). ^a^ Odds ratios compare the relative odds of each category versus all other categories combined; reference category: career firefighters. An OR > 1 indicates higher relative odds in volunteer compared with career firefighters. ^b^ Fisher’s exact test (two-sided). ^c^ Includes 34 responding and 1 returning among volunteer firefighters, and 3 responding and 3 returning among career firefighters. Six volunteer cases in this category occurred specifically during donning of personal protective equipment, all classified as SCA-PNT. ^d^ Haldane–Anscombe continuity correction applied due to a zero cell (no volunteer fatalities during “other duties”).

**Table 3:** Age distribution of line-of-duty firefighter fatalities by employment status.

Age Group (Years)	Volunteer (n = 73)	Career (n = 39)	Total (n = 112)
<20	4 (5.5)	0 (0.0)	4 (3.6)
20–29	9 (12.3)	12 (30.8)	21 (18.8)
30–39	8 (11)	18 (46.2)	26 (23.2)
40–49	14 (19.2)	6 (15.4)	20 (17.9)
50–59	26 (35.2)	3 (7.7)	29 (25.9)
≥60	12 (16.4)	0 (0.0)	12 (16.4)

Values are presented as n (column %). The overall distribution differed significantly between formations (Fisher–Freeman–Halton exact test with Monte Carlo approximation, 50,000 replicates: *p* < 0.001; see Section 2.3.1).

**Table 4:** SCA-PNT versus identifiable external mechanism of death, stratified by age group.

Age Group (Years)	External Mechanism (A–E)	SCA-PNT (F)	Total
<50 years	50 (73.5)	18 (26.5)	68 (100.0)
≥50 years	9 (22.0)	32 (78.0)	41 (100.0)
Total	59 (54.1)	50 (45.9)	109 (100.0)

Values are presented as n (row %). The 50-year cut-point was selected a priori, reflecting the age threshold defined in Polish regulations governing periodic medical examinations of operationally active volunteer firefighters [20]. Category G (other/unspecified, n = 3) was excluded from this comparison due to small numbers and etiological heterogeneity. Comparison of mechanism distribution between age strata: OR for SCA-PNT in the ≥50-year group versus the <50-year group = 9.88 (95% CI 3.96–24.66); Fisher’s exact test, *p* < 0.001; reference category: <50 years (see Section 2.3.1).

**Table 5:** Line-of-duty fatality rates per capita and per deployment, by employment status.

Group	Fatalities (n)	Firefighter-Years	Per-Capita Rate (95% CI) ^a^	Deployments	Per-Deployment Rate (95% CI) ^b^
Career (PSP)	39	899,000	4.34 (3.09–5.93)	46,628,000	0.84 (0.60–1.14)
Volunteer (OSP)	73	8,680,000	0.84 (0.66–1.06)	38,732,000	1.89 (1.48–2.37)
Combined	112	9,579,000	1.17 (0.96–1.41)	85,360,000	1.31 (1.08–1.58)
IRR (PSP vs. OSP)	—	—	5.16 (3.50–7.71) ^c^	—	0.44 (0.30–0.65)

Incidence rate ratio (IRR) for the per-capita comparison (career vs. volunteer, reference category: volunteer firefighters): 5.16 (95% CI 3.50–7.61), *p* < 0.001. Incidence rate ratio (IRR) for the per-deployment comparison (volunteer vs. career, reference category: career firefighters): 2.25 (95% CI 1.53–3.32), *p* < 0.001. ^a^ Per 100,000 firefighter-years. Population denominators: 29,000 career and 280,000 operationally eligible volunteer firefighters, applied as a constant point estimate for the 31-year observation window (see Section 2.3.2). Exact Poisson 95% confidence intervals. ^b^ Per 1,000,000 firefighter-deployments. Total participations of career and volunteer firefighters in fires, non-fire emergencies, and false alarms over the period 1995–2025, retrieved from the annual statistical bulletins of the KG PSP. Exact Poisson 95% confidence intervals. ^c^ IRR confidence intervals computed from the standard error of the log-IRR; *p*-values from the corresponding Wald test (see Section 2.3.2).

## Data Availability

The original contributions presented in this study are included in the article. Further inquiries can be directed to the corresponding author.

## Abbreviations

The following abbreviations are used in this manuscript:

SCA-PNT	Sudden Cardiac Arrest Presumed Non-Traumatic
SCD	Sudden Cardiac Death
FF	Firefighters
KSRG	The National Rescue and Firefighting System
KG PSP	The National Headquarters of the State Fire
PPE	Personal Protective Equipment
NIOSH	National Institute for Occupational Safety and Health
USFA	United States Fire Administration
LODD	Line-of-duty death
